# Roles of Matrix Metalloproteinases and Their Natural Inhibitors in Prostate Cancer Progression

**DOI:** 10.3390/cancers6031298

**Published:** 2014-06-27

**Authors:** Yixuan Gong, Uma D. Chippada-Venkata, William K. Oh

**Affiliations:** Division of Hematology and Medical Oncology, The Tisch Cancer Institute, Icahn School of Medicine at Mount Sinai, New York, NY 10029, USA

**Keywords:** MMP, TIMP, prostate cancer

## Abstract

Matrix metalloproteinases (MMPs), a group of zinc-dependent endopeptidases involved in the degradation of the extracellular matrix, play an important role in tissue remodeling associated with various physiological processes such as morphogenesis, angiogenesis, and tissue repair, as well as pathological processes including cirrhosis, arthritis and cancer. The MMPs are well established as mediators of tumor invasion and metastasis by breaking down connective tissue barriers. Although there has been a vast amount of literature on the role of MMPs in invasion, metastasis and angiogenesis of various cancers, the role of these endopeptidases in prostate cancer progression has not been systematically reviewed. This overview summarizes findings on the tissue and blood expression of MMPs, their function, regulation and prognostic implication in human prostate cancer, with a focus on MMP-2, -7, -9, MT1-MMP and tissue inhibitor of metalloproteinase 1 (TIMP-1). This review also summarizes the efficacy and failure of early-generation matrix metalloproteinase inhibitors (MMPIs) in the treatment of metastatic prostate cancer and highlights the lessons and challenges for next generation MMPIs.

## 1. Introduction

Prostate cancer is the most frequently diagnosed cancer in American men, accounting for 27% of new cancer diagnoses. In 2014, there will be an estimated 233,000 new cases and 29,480 prostate cancer-related deaths [1]. With the emergence of new treatment options, and thus better therapeutic strategies, mortality rates of prostate cancer have been significantly reduced in the past decade. New oral agents such as abiraterone acetate (an adrenal androgen synthesis inhibitor) and enzalutamide (an androgen receptor signaling inhibitor) are now available to inhibit the androgen receptor (AR) pathway that remains activated by substantial residual androgen levels in castration-resistant prostate cancer (CRPC) tissue, despite androgen deprivation therapy [2]. With the addition of other promising treatments including cabazitaxel, a chemotherapeutic agent [3], sipuleucel-T, a novel autologous cell-based immunotherapy [4], and most recently, radium-223 (^223^Ra), a first in class α-emitting radiopharmaceuticals [5,6], the prospect for improving overall survival of men with metastatic CRPC has improved significantly. Despite this increased armamentarium of treatment options, progress in developing tools that predict who will benefit from each therapy has been slow. Currently there is a lack of biomarkers for treatment selection and response. With increased understanding of the biology of this disease, and further improvement of predictive tools with the inclusion of biomarkers, patient management strategies will be further improved in the future, with patient stratification to enrich for populations who may be most likely to benefit from each specific treatment. 

## 2. Overview of Metalloproteinases and Their Natural Inhibitors

### 2.1. Structure and Classification of MMPs

**Figure 1:**
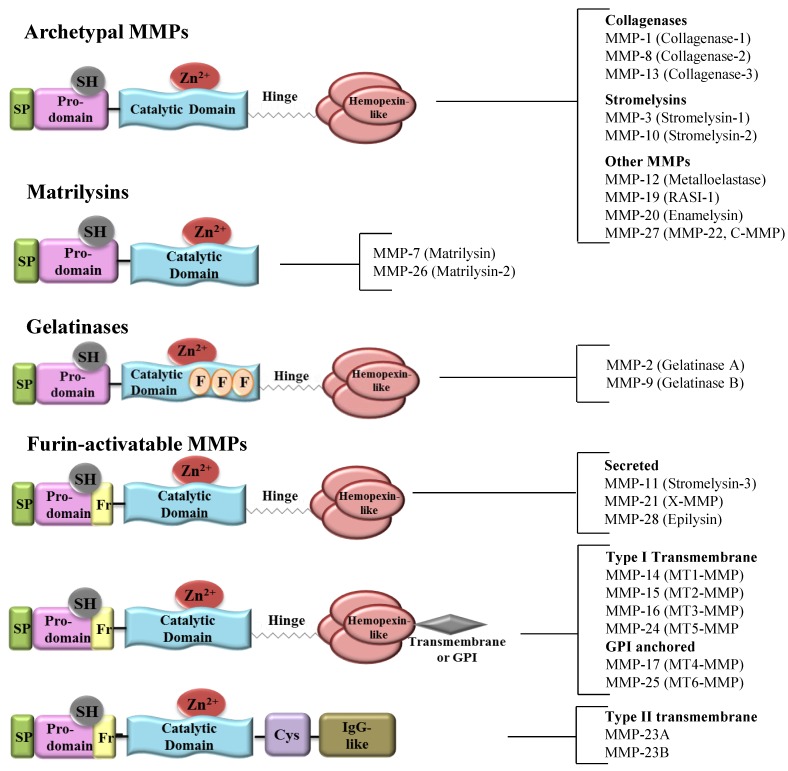
Domain structure classification of Mammalian MMPs. Structurally, MMPs are classified into four groups namely archetypal MMPs, matrilysins, gelatinases and furin-activatable MMPs. Their typical structure consists of a signal peptide (SP), a prodomain, a catalytic domain, a hinge region, and hemopexin like-domain. The archetypal MMPs are further subdivided into collagenases, stromelysins and others based on their substrate specificities. Matrilysins lack the hemopexin domain. Gelatinases have fibronectin repeats (F) in their catalytic domain. Furin-activatable MMPs contain a furin (Fr) recognition motif and include three secreted, six membrane type (MT) and two unusual type II transmembrane MMPs. Based on the type of attachment to the plasma membrane, MT-MMPs are divided into type I transmembrane MMPs and glycophosphatidylinositol (GPI) MMPs. Type II transmembrane MMPs lack the cysteine switch motif in the prodomain as well as the hemopexin domain, instead have a cysteine array (Cys) followed by an immunoglobulin-like (IgG-like) domain; SH-thiol group.

It is now well-established that tumor initiation, progression and invasion are a consequence of a complex cross-talk between different cell types within the tumor microenvironment. A defining characteristic of malignant tumors is their ability to destroy matrix barriers, permitting invasion into the surrounding connective tissues, intravasation and extravasation, and metastasis to distant organs [7,8]. This process requires the unique action of proteolytic systems responsible for the hydrolysis of basic components of the extracellular matrix (ECM) to facilitate tumor cell dissemination. Although many proteases have been associated with cancer dissemination, a specific group of 24 enzymes, collectively called matrix metalloproteinases (MMPs), has been the focus of much anticancer research [9]. These enzymes are named for their dependence on metal ions for catalytic activity and their potent ability to degrade structural proteins of the ECM [10]. A typical MMP has a multi-domain structure which includes a signal peptide domain, a pro-peptide domain and a catalytic domain, as illustrated in Figure 1. 

### 2.2. Function of MMPs in Tumor Progression

MMPs play a critical role in the classic hallmarks of cancer [11], including migration, invasion, metastasis and angiogenesis (see reference [12] for a review of MMP function in cancer).

Unquestionably, these are a result of the autocrine and paracrine cross-talk between tumor cells and surrounding stromal cells in the tumor microenvironment, including endothelial cells, ﬁbroblasts, macrophages, mast cells, neutrophils, *etc*., involving regulation of multiple growth factors and cytokines [13].

Based on *in vitro* and animal studies, the assumption that MMP overexpression facilitated tumor progression prompted the development of MMP inhibitors (MMPIs) as cancer therapeutics. However, multiple disappointing clinical trials prompted serious reevaluation of MMP inhibition strategies because, while these inhibitors could be potential anti-cancer agents, they might also interfere with normal development and host defense processes [9,14]. It is now realized that MMPs can also be protective and beneficial; thus a better understanding of MMP functions in specific cellular contexts will result in improved targeting strategies against detrimental effects of MMPs. 

### 2.3. Regulation of MMP Activities

With the ability to control cell fate and alter developmental and pathological outcomes, there is a need for greater regulation. MMP activities are tightly regulated at levels of mRNA transcription [15,16] and stability control [17], at the protein level via their activators [18,19] and inhibitors [20], and through cellular compartmentalization [21]. Furthermore, individual MMPs are separately regulated, with their expression highly tissue-specific with a broad range of preferred substrates. Knowledge of the mechanisms by which MMP expression or activity is regulated is of interest because of the potential therapeutic applications of manipulating such processes, as well as enhancing our understanding of the basic mechanisms of tumor invasion and metastasis [22].

### 2.4. Natural MMP Inhibitors: Tissue Inhibitors of Metalloproteinase (TIMPs)

MMPs are specifically inhibited by a family of small extracellular proteins known as the tissue inhibitors of metalloproteinases (TIMPs). There are four members of the TIMP family, TIMP-1, -2, -3 and -4, each inhibiting the activities of various MMPs with varying efficiency (reviewed in [23]). TIMPs form tight 1:1 stoichiometric complexes rather than covalent bonds with MMPs with inhibition constants in the subnanomolar range [20]. As key regulators of MMPs, TIMPs play a pivotal role in determining the influence of the extracellular matrix, of cell adhesion molecules, and of many cytokines, chemokines and growth factors on cell phenotype in various physiological and pathological conditions [20]. In addition, there is evidence that TIMPs have biological activities independent of metalloproteinase inhibition including effects on cell growth, differentiation, cell migration, apoptosis, and angiogenesis [24]. In summary, the pleiotropic effects of the TIMP family are profound and numerous, the underlying biology of which is not completely understood.

## 3. Function and Regulation of MMPs in Prostate Cancer

**Figure 2:**
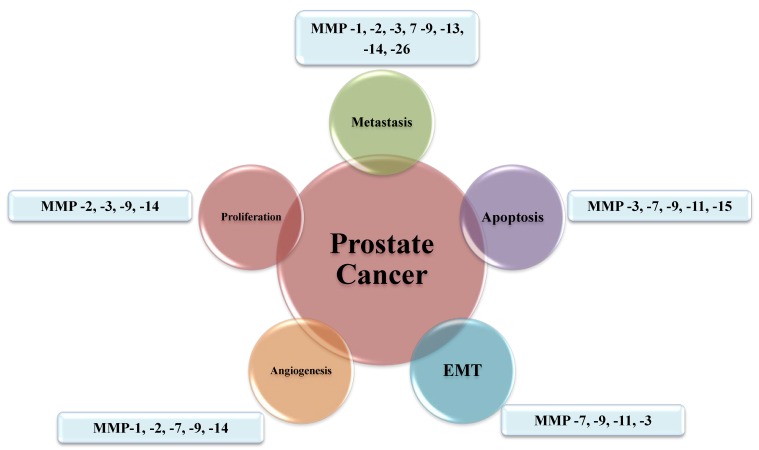
Roles of MMPs in several hallmarks of prostate cancer progression.

In prostate cancer tissue, there is an imbalanced expression of MMPs and TIMPs, manifested as a general loss of TIMPs and an upregulation of MMPs. Elevated MMP activity promotes prostate cancer progression not only by facilitating metastasis, but also by profoundly impacting multiple steps of cell proliferation, apoptosis, angiogenesis and epithelial to mesenchymal transition (EMT). As such, it is generally thought that MMPs are more active in advanced stages of prostate cancer, as indicated by the fact that most MMPs display higher expression in cancers with high Gleason scores (Table 1). Analysis of MMP mRNA and protein levels in the serum and tissue samples from prostate cancer patients has shown that increased expression of MMP-2, -3, -7, -9, -13, -14, -15 and -26 is correlated with advanced or metastatic disease, while MMP-1 expression is associated with lower grade tumors and a lower incidence of invasion (see Table 1 for a summary of literature). The particular roles these MMPs play in the hallmarks of cancer progression are illustrated in Figure 2. Among the diverse members of the homologous MMP family, MMP-2, -7, -9 and MT1-MMP are the most well studied for their roles in prostate cancer progression and thus will be the focus of this review. Overall, expression of these MMPs promote prostate cancer progression but with subtle differences in their pattern of expression, biological function and regulation and prognostic value. For instance, in genetically-engineered mice, although the lack of MMP-2, -7, or -9 in CR2-Tag mice all led to reduced tumor vascularity, the loss of MMP-2 conferred decreased lung metastasis and increased survival, while the lack of MMP-9 led to increased perivascular invasion [25]. This observation highlights that there is overlapping as well as unique and even opposing functions between the diverse members of the MMP family in cancer.

**Table 1:** Characterization of MMP and TIMP expression in human prostate cancer.

MMPs	Methods		Conclusion	References
MMP-3 & TIMP-1	ELISA		↑TIMP-1 and MMP-3 in the serum of PCa patients with metastases	[26]
MMP-1	IHC		↑in lower grade tumor and lower incidence of invasion	[27]
MMP-2	ISH and Northern		↑MMP-2/TIMP-1 in the high stage tumors	[28]
MMP-2,-9 & TIMP-1,-2	ISH		↑MMP-2, -9, ↓TIMPs in higher tumor stage; MMP-2 and TIMP-1 are independent predictors of outcome	[29]
MMP-2	IHC		↑MMP-2 in varying Gleason grades of malignant prostate cancer	[30]
MMP-2	ELISA		↑serum MMP-2 correlated well with the clinical course of prostate cancer with bone metastasis	[31]
MMP-2	IHC		↑MMP-2 in higher Gleason score tumor and in lymph node metastases	[32,33]
MMP-7 & TIMP-1	Northern		↑MMP-7 and MMP-7/TIMP-1 in advanced prostate carcinoma	[34]
MMP-9	Zymography		↑MMP activity in malignant prostatic tissue compared with benign prostate hyperplasia	[35]
MMP-2,-9 & -13	ELISA		↑plasma MMP-2, MMP-9 and MMP-13 in PCa patients with metastasis	[36]
MMP-2 & MT1-MMP	IHC, Western & zymography		↑MT1-MMP in secretory cells; heterogeneous MMP-2 and MT1-MMP staining within the epithelial components of the cancer glands	[37]
MMP-15 & -26	qRT-PCR	↑MMP-15 and MMP-26 correlated positively with Gleason score		[38]
MMP-2	IHC and ISH	↑MMP-2 in dysplastic epithelium and prostatic adenocarcinoma		[39]
MMP-2 & -9	ISH	↑MMP-2&-9 associated with the Gleason score of the tumors		[40]
MMP-2 & TIMP-2	IHC	↑MMP-2&TIMP-2 co-expressed in adenocarcinomas and correlated with prognostic variables		[41]
MMP-2,-9 & TIMP-1,-2	IHC	↑MMP-2,↓MMP-9,↓TIMP-1 in malignant tumors; ↓TIMP-2 in the stroma cells surrounding the tumor		[42]
MMP-1,MT1-MMP, MMP-7 & -9	IHC	↓MMP-1&↑ MT1-MMP, MMP-7 & MMP-9		[43]
MMP-2	IHC	↑MMP-2 in CTC associated with high grade tumors in metastatic disease		[44]
MMP-2,-3,-9,-10 & -13	IHC	↑MMP-2,-3&-10 in neoplastically transformed cells; no immunoreactivity was observed against MMP-9 and -13		[45]
MMP-9	IHC	↑MMP-9 in high grade tumors strongly associated with high Gleason score		[46]
MMP-2,-7 & -9	IHC&ISH	↑MMP-2&-7 in tumors and location of these MMPs varied		[47]
MMP-7	ELISA	↑serum MMP-7 concentration was significantly elevated in patients with distant metastasis		[48]
TIMP-1	ELISA	↑plasma TIMP-1 associated with poorer survival in CRPC		[49]
MMP-2	IHC	MMP-2 expression by >50% of malignant epithelial cells was associated with decreased disease-free survival		[50]
MMP-2,-3,-7,-9,-13 & -19	IHC	↑MMP-9 expression associated with recurrence-free and disease-specific survival in organ confined PCa		[51]
MMP-9	qRT-PCR	↑MMP-9 related to biochemical recurrence		[52]
MMP-2 & -9	ELISA	↑serum MMP-9 in PCa patients, but not associated with bone metastasis		[53]
MMP-2,MMP-9 & MMP-9/NGAL	Chromatography, zymography&mass spectrometry	↑MMP-9 and dimer in urine from prostate and bladder cancer groups		[54]
MMP-2 & -9	IHC	MMP-2&-9 significantly associated with several conventional prognostic factors		[55]
MMP-7	Northern, ISH	↑MMP-7 in the epithelial cells of primary prostate adenocarcinoma as well as metastatic cells		[56]

IHC: immunohistochemistry; ISH: *in situ* hybridization.

### 3.1. MMP-2

**Figure 3:**
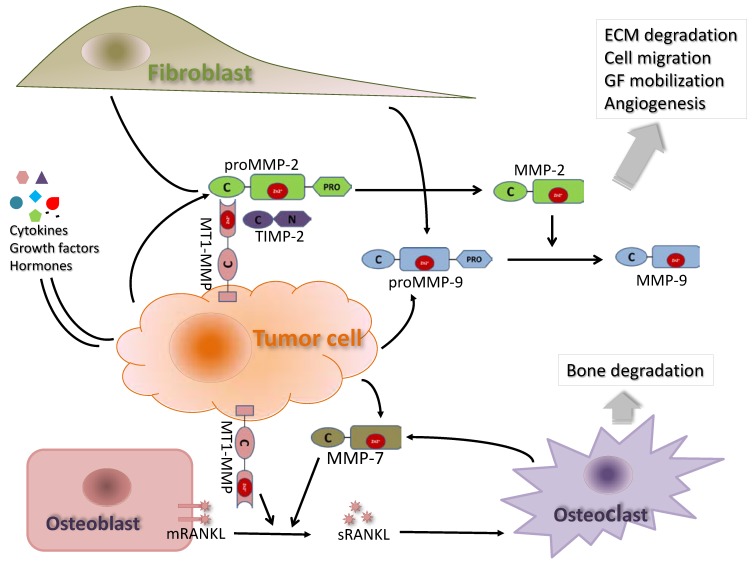
Illustration of key interactions between MMP-2, -7, -9 and -14 and their proposed roles in prostate cancer progression. MMP-2 and MMP-9 are secreted as pro-enzymes by both tumor cells and fibroblast cells in the tumor microenvironment. MT1-MMP (MMP-14) specifically activates the latent proMMP-2 on the tumor cell surface through the formation of a complex with TIMP-2. Activated MMP-2 can activate other proMMPs such as proMMP-9 through enzymatic cleavage. MT1-MMP is expressed and anchored on the membrane of tumor cells. MMP-7 is released by tumor cells as well osteoclast cells in the bone. MT1-MMP and MMP-7 can cleave membrane bound receptor activator of NF-κB ligand (mRANKL) on the osteoblast surface and the resulting soluble RANKL (sRANKL) mediates the activation of osteoclasts at or near the tumor-bone interface, resulting in bone degradation.

*Expression pattern*: Both MMP-2 (gelatinase A) and MMP-9 (gelatinase B) belong to the gelatinase subfamily, which is a group of proteolytic enzymes distinguished by their fibronectin-like gelatin-binding domain (Figure 1) and their ability to degrade gelatin into its sub-compounds (polypeptides, peptides, and amino acids) [57]. Besides its direct proteolytic actions on the ECM, MMP-2 also activates other cellular substrates such as FGFR and MMP-9 through enzymatic cleavage [58,59] (Figure 3). Increased expression of MMP-2 has been extensively reported in prostate cancer [28,29,30,32,33,39,40,41,56] and higher MMP-2 expression has been correlated with larger tumor size, higher Gleason score and more advanced pathological TNM stage (a clinical cancer staging system used to describe the extent of a person’s cancer) [32,40,41,42]. In a recent study, Murray et al. showed that MMP-2 expression was not present in micrometastasis and surrounding stromal cells of low grade tumors, but was present in metastatic disease, strongly suggesting that increased MMP-2 expression was associated with prostate cancer progression and metastasis [44].

Although immunohistochemistry staining and in situ hybridization have consistently demonstrated increased expression of MMP-2 in prostate cancer tissues, studies of MMP-2 expression in cultured prostate cancer cells have given somewhat inconsistent results. In a study by Lokeshwar *et al*. conditioned media from freshly cultured malignant prostate explants contained a higher proportion of the active form of MMP-2 than normal tissues [60]. However, in another study to examine MMP-2 secretion from cultured normal and neoplastic prostate cells derived from different zones of the prostate, only prostate stromal cells secreted the pro-enzyme form of MMP-2, whereas conditioned media from epithelial cells of various origins demonstrated little to no pro-MMP-2 as examined by zymography [61]. The absence of MMP-2 expression in tumor epithelial cells in the study can be potentially explained by the low-grade tumor samples used in the study or lack of stromal support in cell culture. It was demonstrated that addition of fibronectin to cell culture induced high expression of pro and active forms of MMP-2 in prostate cancer cell lines [62], suggesting that a cell culture model that more closely mimics the *in vivo* tumor microenvironment is critical when studying MMP expression and function *in vitro*.

*Function*: Due to a lack of highly selective MMP inhibitors, knowledge on the function of individual MMPs has mostly come from overexpression or knockdown studies. Although most of these studies were carried out in non-prostate cancer models, it is widely accepted that MMP-2 is important in the dissemination and invasion of various cancer cells and activation of angiogenesis. Since MMP-2 can be released from malignant cells or surrounding stromal components, the relative contribution of MMP-2 secretion from each compartment to tumor progression has been studied. It was shown that suppression of MMP-2 activity by antisense oligonucleotides in chondrosarcoma cells resulted in suppression of tumor growth in a mouse host with wild-type MMP-2 through reduced angiogenesis [63]. In a complementary study, MMP-2 wild-type B16-BL6 melanoma cells implanted into MMP-2 deficient host mice also exhibited slightly decreased tumor growth and a significant delay in lung colonization after intravenous injection [64]. These studies highlight the importance of MMP-2 derived from both tumor and tumor microenvironment in tumor metastasis and angiogenesis.

*Regulation*: MMP-2 activity is regulated at multiple steps including mRNA transcription, post-translational activation by membrane type-1 MMP (MT1-MMP) and inhibition of protease activity by its natural inhibitors (TIMPs). Members of the MMP family have a similar gene promoter organization, resulting in similarity on regulation of gene transcription. Pathways activating AP-1, AP-2, Sp-1, ETS and NF-κB transcriptional factors have been implicated in the regulation of MMP-2 mRNA expression [65]. WIF1, a naturally-occurring Wnt inhibitor, suppressed invasive capacity of PC3 cells via down-regulation of MMP-2 and -9 activities [66], indicating that Wnt/β catenin pathway is an important player in the regulation of MMPs in human prostate. SIRT1 (a nicotinamide adenine dinucleotide-dependent histone deacetylase) has been reported as a positive regulator of MMP-2 activity by promoting its expression, stability and activity [67]. At the post-translational level, MT1-MMP specifically activates the latent pro-MMP-2 on the tumor cell surface through the formation of a complex with TIMP-2 [68,69] (Figure 3).

As a major protease involved in modifying the microenvironment, MMP-2 is reciprocally regulated by various growth factors, hormones, cytokines and enzymes commonly found in the extracellular space. For instance, addition of fibronectin into common culture media induced high expression levels of pro and active forms of MMP-2 in several prostate cancer cell lines [62]. Activation of protease-activated receptor 1 (PAR1) and PAR2 by activating peptides increased expression of MMP-2 and -9 in prostate cancer cell lines [70]. Treatment of prostate stromal cells with TGF-β moderately increased secretion of pro-MMP-2 protein, while treatment of prostate epithelial cells with TGF-β induced expression and secretion of both MMP-2 and -9, suggesting the critical role of TGF-β contributing to the elevated levels of MMP-2 and -9 observed in prostate cancer [71]. Insulin-like growth factor (IGF)-1 was previously shown to up-regulate MMP-2 production in lung cancer [72], whereas the interleukin (IL)-10/IL-10 receptor axis was found to down-regulate MMP-2 synthesis in prostate cancer cell line PC3 ML [73]. In a follow-up study, IL-10 blocked IGF-1-induced MMP-2 mRNA expression and protein synthesis in primary prostate cells, implying that regulation of MMP expression in prostate cancer cells is a complex interaction of various cytokines present in the tumor microenvironment [57]. In addition, it was shown that ADAM17 (also known as tumor necrosis factor-α converting enzyme; TACE), an enzyme involved in proteolytic ectodomain shedding of cell surface molecules and cytokines, contributed to androgen-independent prostate cancer cell invasion by shedding of EGFR ligand TGF-α, which subsequently activated the EGFR-MEK-ERK signaling pathway, finally leading to overexpression of MMP-2 and -9 [74].

In hormone-sensitive prostate cancer cells, MMP-2 expression is positively regulated by activation of the androgen pathway. R1881 (a synthetic androgen) increased pro-MMP-2 expression in androgen-sensitive LNCaP and LAPC-4 cell lines in a PI3 kinase-dependent manner, which was blocked by the androgen antagonist bicalutamide [75]. Two putative androgen response element (ARE) motifs on the MMP-2 promoter were required for androgen-stimulated MMP-2 expression [76]. Accumulating evidence also suggests an enhancing effect of estrogens on prostate cancer progression through regulation of MMP-2 expression. Conditioned media collected from an estradiol-treated immortalized prostatic stromal cell line WPMY-1 and primary stromal cells promoted invasion of prostate cancer cells in a paracrine mechanism by up-regulating MMP-2 expression at both mRNA and protein levels. Mechanistic studies showed that estradiol-induced TGF-β1 expression was involved in the stimulation of MMP-2 expression in prostatic stromal cells [77].

In addition to growth factors, hormones and cytokines, some phytochemicals and therapeutic agents can also affect MMP-2 expression. Genistein, a phytoestrogen belonging to the family of isoflavones, inhibited cell invasion of prostate cancer cell lines by blocking MMP-2 activity [78]. The effect was seen at concentrations of genistein physiologically achievable in the blood after dietary consumption, indicating the potential impact of dietary compounds on prostate cancer progression via targeting MMP-related processes. The 5-α-reductase inhibitors (5-ARIs), finasteride and dutasteride, have been used as treatments for benign prostatic hyperplasia and as potential chemopreventive agents for prostate cancer. Although there is a debate on increased prevalence of high-grade tumors among 5-ARI-treated patients [79], a recent study showed that finasteride may attenuate tumor aggressiveness and invasion through MMP-2 and MMP-9 downregulation in prostate cancer cells [80].

### 3.2. MMP-9

*Expression pattern*: Matrix metalloproteinase-9 (MMP-9, gelatinase B) is also produced in an inactive form (pro-MMP-9) that needs to be activated by other members of the MMP family such as MMP-2 and -3 [81] (Figure 3). There are discrepancies in reports of the expression of MMP-9 in prostate cancer tissue. Protein expression has been reported to be either absent [45,71] or present [40,46] and the localization of MMP-9 expression reported in the literature also varies. In some studies, expression of MMP-9 mRNA was detected only in macrophages in areas of prostatic inflammation [47] or in the invasive edge of higher Gleason score tumors [40]. In contrast, Trudel *et al*. reported that 94.1% of prostate cancer cells actually exp0ressed MMP-9 in the cytosol and intracellular MMP-9 expression was directly correlated with Gleason score, but not prognosis [46]. The discrepancies in MMP-9 expression could be partly explained by differences in the degree of invasiveness of the tumor samples used in the studies or by the sensitivity of detection methods. Nonetheless, it is interesting to note that in a study of fresh prostate tissue obtained from 22 radical prostatectomies, the overall collagenolytic and gelatinolytic activities was relatively low in comparison to other malignancies such as basal cell carcinomas, which may help explain why the majority of localized primary prostate tumors remain confined to the prostate for relative long periods of time compared to other more invasive cancers [82].

*Function*: Like MMP-2, MMP-9 derived from both tumor cells and tumor microenvironment plays important roles in the process of cancer metastasis. The latter was clearly demonstrated by an experiment in immunocompromised mice in which host MMP-9 status significantly affected growth of transplanted osteolytic/osteogenic-inducing prostate adenocarcinoma in the calvaria [77].

Altered MMP-9 expression on the cell surface and in its secreted forms is thought to contribute to enhancement of prostate cancer growth, metastasis and angiogenesis. LNCaP, DU-145, and PC-3 are commonly used prostate cancer cell lines that have demonstrated low, moderate, and high metastatic potential in Matrigel invasion assays, respectively [83,84]. PC-3 cells show increased expression of MMP-9 compared with LNCaP and DU-145, which correlate with the highest invasive activity among the cell lines [84]. Stable expression of human MMP-9 in poorly metastatic LNCaP cells produced a 2 to 3-fold increase in MMP-9 activity with a concomitant increase in invasiveness [85]. SiRNA-mediated silencing of MMP-9 inhibited Matrigel invasion and *in vitro* angiogenesis, and induced apoptosis in DU145 and PC3 cells [86,87]. MMP-9 is involved in the regulation of angiogenesis; antisense ablation of MMP-9 expression in DU-145 and PC-3 cells produced concomitant inhibition of the gene expression of the proangiogenic factors such as vascular endothelial growth factor (VEGF) and intercellular adhesion molecule-1 (ICAM-1). MMP-9 knockdown also increased the release of angiostatin, a key protein that suppresses angiogenesis and decreases secretion of VEGF, the most common and potent angiogenic factor, in PC-3 cells [88]. Furthermore, MMP-9 can also activate urokinase plasminogen activator (uPA), serpin protease nexin-1 (PN-1) and other matrix proteins involved in the process of invasion and angiogenesis [89,90].

*Regulation*: Tumor-stromal interactions regulate MMP-9 expression and their function in prostate cancer. Co-culture of prostate cancer and stromal cells *in vitro* enhanced expression of pro-MMP-9 in prostate cancer cells, and down-regulated TIMPs in stromal cells. MMP-9 expression was also induced in metastatic PC-3 cells grown in human fetal bone implants in severe combined immunodeficient (SCID) mice [91]. Co-culture of endothelial cells with prostate cancer cells also significantly enhanced expression of MMP-9 and subsequent invasiveness of cancer cells through increased IL-6 secretion from endothelial cells [92], suggesting that growth factors or cytokines secreted by tumor cells, stromal cells, and infiltrating inflammatory cells in the tumor microenvironment collectively regulate MMP-9 gene expression in an autocrine and paracrine manner. Specifically, several cytokines and related proteins can regulate MMP-9 expression in prostate cancer. It was shown that increased IL-6 expression, which is often seen in advanced prostate cancer [93], resulted in activation of MMP-9 expression through the TGF-β pathway [92]. Poorly metastatic PC-3P cells overexpressing IL-8 displayed up-regulated MMP-9 mRNA and collagenase activity in vitro, resulting in increased invasion through Matrigel [94]. CXC chemokine receptor-4 (CXCR4) plays an important role in prostate cancer metastasis through the up-regulation of VEGF and MMP-9 both *in vitro* and *in vivo* [95]. Bombesin, a neuropeptide hormone present in prostatic adenocarcinomas, stimulated secretion of MMP-9 in human prostate cancer cell lines [96]. In tumor tissue, expression of MMP-9 and bombesin was observed in almost the same population of cancer cells and was associated with high grade tumors [97]. Fibroblast growth factor-inducible 14 (Fn14), a transmembrane receptor binding to TWEAK, promoted androgen-independent prostate cancer progression through MMP-9 and correlated with poor treatment outcome [98]. Last but not least, it was shown that increased MMP-9 expression was associated with the loss of PDEF (prostate-derived ETS factor) in more aggressive prostate cancers. Studies showed that PDEF suppressed MMP-9 mRNA expression and resulted in decreased colony formation, cell migration, and cellular invasiveness in prostate cancer cells [99].

Unlike MMP-2, MMP-9 expression appears to be negatively regulated by the androgen pathway [100]. Androgen supplementation significantly reduced secretion and activity of MMP-9 in AR-positive prostate cancer cells grown in androgen-depleted media [101]. Conversely, flutamide administration resulted in a marked increase in expression of MMP-9 in experimental rats, which could be reversed by oral administration of daidzein, an isoflavone compound [102]. Since isoflavones are metabolized differently in rodents in comparison to humans, the relevance of the latter observation in human setting is unclear. In an interesting recent publication, the authors compared the effects of various androgen deprivation therapies on prostate cancer metastasis *in vitro* and *in vivo*. Results showed that the anti-androgens, bicalutamide and enzalutamide, suppressed prostate cancer cell growth and yet significantly enhanced prostate cancer cell invasion through enhancement of the TGF-β1/Smad3/MMP-9 pathway; whereas the newer anti-AR compounds, ASC-J9 and cryptotanshinone suppressed both prostate cancer cell growth and cell invasion in cell culture and *in vivo* models through down-regulation of MMP-9 expression [103]. These results indicated that novel androgen therapies that target both AR and TGF-β1/Smad3/MMP-9 pathways may be better at treating metastatic prostate cancer in the castration-resistant setting by suppressing the potential risk of increased MMP-9 expression and promotion of cancer metastasis.

### 3.3. MMP-7

MMP-7 (matrilysin, pump-1) is the smallest known member of the MMP family, as seen from the structure noted in Figure 1. MMP-7 is secreted as a 28 kDa proenzyme which can be activated *in vitro* by APMA, trypsin and high temperatures and *in vivo* by MMP-3 to a 18 kDa active MMP-7 enzyme [104]. Activated MMP-7 mediates the cleavage of ECM and basement membrane proteins such as fibronectin, collagen type IV, laminin, and others, as well as mediates the ectodomain shedding of pro- and anti-tumor molecules such as tumor necrosis factor-α, Fas ligand, heparin-binding epidermal growth factor, E-cadherin and β4-integrin [105,106,107]. Furthermore, MMP-7 can serve as a double-edged sword to regulate angiogenesis not only by mobilizing endogenous pro-angiogenic factors but also by generating angiogenic inhibitors such as endostatin [108]. Elevated MMP-7 expression has been demonstrated in a variety of epithelial and mesenchymal tumors [109,110,111].

In prostate cancer, 77% and 50% of prostate tumors were found to focally express MMP-7 by *in situ* hybridization analyses and western blotting, respectively [47]. In a study aimed at investigating serum levels of various MMPs in the prostate cancer, circulating MMP-7 was significantly elevated in individuals with distant metastases, suggesting that MMP-7 may play a role facilitating distant metastases [48]. Prostate cancer is characterized by the recurrent translocation and amplification of ETS transcription factors [112]. It was reported that the ETS family transcription factors, E1AF and ETV1 (ETS-related 81), increased expression of MMP-7 and contributed to tumor aggression of prostate cancer [113,114]. MMP7 was also elevated in mouse prostates following activation of the nuclear β-catenin pathway [115].

A recent publication has linked MMP-7 to bone metastasis from prostatic adenocarcinoma. Prostate and breast cancers are unique among solid tumors in their strong propensity to metastasize to bone [116,117]. Up to 84% or more of prostate cancer patients have bone metastases at autopsy [117]. Among all MMPs that are highly expressed in the tumor-bone microenvironment (MMP-2, -3, -7, -9, and -13), only osteoclast-derived MMP-7 significantly contributed to human breast-to-bone metastatic tumor growth and tumor-induced osteolysis in experimental mice [118]. Mechanistically, it is proposed that MMP-7 secreted by both osteoclasts and tumor cells cleaves membrane bound receptor activator of NF-κB ligand (RANKL) on the osteoblast surface and the resulting soluble RANKL mediates the activation of osteoclasts at or near the tumor-bone interface, resulting in bone degradation [119] (Figure 3).

### 3.4. MMP-14 (MT1-MMP)

Of six MT-MMPs that have been identified, type 1 (MT1-MMP) has been the best studied in prostate cancer. MT1-MMP shares many conserved structural features with other MMPs, but differs in that it is anchored to the plasma membrane by a transmembrane domain while exposing its catalytic domain on the surface of the cells [120] (Figure 1). MT1-MMP has a broad repertoire of ECM substrates including collagen, laminin, fibronectin, and vitronectin and thus plays a major role in degrading the extracellular matrix to clear a path facilitating cell migration and invasion [121,122]. It also indirectly is involved in ECM remodeling by activating pro-MMP2, as described previously [68,69] (Figure 3). Clinically, higher MT1-MMP mRNA levels were seen in prostatic intraepithelial neoplasia and prostate cancer than in benign epithelial tissue [43].

The activity of MT1-MMP can be regulated at multiple levels: transcription controlled by pathways such as the Wnt-β-catenin signaling [123,124], protein trafficking between the cytoplasm and plasma membrane [125,126], and binding to its endogenous inhibitors, TIMP-2 and TIMP-3 [127]. In prostate cancer, additional pathways have been shown to regulate MT1-MMP expression. Synthetic androgen and IGF-1 treatment increased MT1-MMP expression in LNCaP cells and inhibition of IGF-1R in PC-3N cells decreased MT1-MMP expression, highlighting the role of the IGF-1R pathway in regulating MT1-MMP expression [128]. LIM kinase 1 (LIMK1), an actin and microtubule cytoskeleton modulatory protein overexpressed in a number of cancerous tissues and cells, increased expression of MT1-MMP, transcriptional activation and its localization to the plasma membrane in prostate cancer cells. Increased expression of both MT1-MMP and LIMK1 has been noted in prostate tumor tissues [129].

In prostate cancer cell lines, increased MT1-MMP expression has been associated with increased aggressiveness [130] and increased transition from androgen-dependent to independent growth [131,132]. Mechanistically, prostate cancer cells with high levels of MT1-MMP showed an increased ability to degrade and invade Ln-10 barriers to facilitate invasion [133]. In a 3-D cell culture model, transfection of LNCaP cells with MT1-MMP induced epithelial-to-mesenchymal transition by decreasing epithelial markers and enhancing mesenchymal marker expression through upregulation of Wnt5a [63]. An interesting recent study showed that overexpression of MT1-MMP in LNCaP cells promoted a more aggressive phenotype by eliciting oxidative stress in prostate cancer cells, which required adhesion to ECM proteins and was impeded by anti-β1 integrin antibodies [134]. This study highlights a novel mechanism to link MMP proteolytic activity to the induction of a more invasive phenotype through an oxidative stress-related mechanism.

Since approximately 90% of hematogenous metastases in prostate cancer occurs in bone, MT1-MMP is an especially important enzyme due to its collagen degrading activity [135]. The role of MT1-MMP in prostate cancer metastasis was clearly demonstrated in a study by Bonfil *et al*. in which LNCaP cells overexpressing MT1-MMP produced larger tumors and more osteolysis in bone compared to control cells, whereas DU145 cells with MT1-MMP knockdown induced osteogenic changes only [135]. Mechanistically, MT1-MMP not only played an essential role in bone matrix degradation, but also functioned as a sheddase to release RANKL, an osteoclastogenic factor, from the surface of osteoblasts and prostate cancer cells to enhance osteoclast differentiation and activate Src-dependent prostate cancer cell migration [136] (Figure 3).

### 3.5. Tissue Inhibitor of Metalloproteinase 1 (TIMP-1)

TIMPs are secreted by both epithelial and stromal cells, and unregulated TIMP expression has been implicated in tumor invasion and metastasis. Biochemical analysis has shown that significant amounts of TIMP protein are secreted by normal prostate tissue, but that this expression is either markedly reduced or not detectable in conditioned media from neoplastic tissues [60]. *In situ* hybridization of tumor tissues demonstrated that TIMP-1 and TIMP-2 were expressed at elevated levels in the stroma of low Gleason score tumors, but were negative in higher Gleason score tissues (GS 8–10) [29]. Furthermore, TIMP-1 and TIMP-2 expression were high in organ-confined specimens, but lower or negative in locally advanced tumors with capsular penetration, positive surgical margins, seminal vesicle involvement and/or lymph node invasion. In summary, decreased TIMP-1 expression in prostate tumor tissues is consistent with the function of the protein as an MMP inhibitor. Nevertheless, circulating TIMP-1 levels in the blood do not seem to follow the same pattern of expression in the tissue and appear to be paradoxically up-regulated in prostate cancer patients instead (see more details in Section 3.1).

TIMP-1 is roles in inhibiting tumor cell invasion, as illustrated in multiple studies [137,138,139]. However a 28.5 kDa secreted glycoprotein that shares 40% and 28% amino acid sequence homology with its family partners TIMP-2 [140] and TIMP-3 [100], respectively. Due to its MMP inhibitory activity, TIMP-1 was initially thought to have major, more recent studies have demonstrated that TIMP-1 may also promote tumor growth in an MMP-independent manner by stimulating cancer cell growth and inhibiting apoptosis [141,142,143,144,145,146]. Consistent with this pro-tumorigenic role of TIMP-1, several clinical studies have demonstrated that elevated TIMP-1 levels in tumor tissue or peripheral blood are associated with poor clinical outcomes in a range of malignancies [147,148,149,150,151,152,153,154]. Recently, our group reported that prostate cancer patients showed significantly elevated circulating TIMP-1 protein levels compared to men without cancer [155] and that elevated plasma TIMP-1 levels predicted a poor survival in CRPC patients [49,156]. However, it is not entirely understood why TIMP-1 was elevated in CRPC and how elevated circulating TIMP-1 contributed to a poor survival. In one study, our group reported that increased TIMP-1 expression modified the tumor microenvironment in favor of cancer progression by stimulating accumulation of cancer-associated fibroblasts (CAFs) within prostate cancer tissues, and that TIMP-1 enhanced prostate CAF proliferation and migration in vitro while promoting ERK1/2 kinase activation in these CAF cells [155]. In a mouse lymphoma model, Gilbert *et al.* showed that TIMP-1 was released in the thymus in response to chemotherapy-induced DNA damage, creating a “chemo-resistant niche” that promoted the survival of minimal residual tumor cells and served as a reservoir for eventual tumor relapse [157]. Further studies are being carried out to fully understand the biological function of this protein in prostate cancer.

## 4. Clinical Implications of MMPs and TIMP-1 in Prostate Cancer

### 4.1. MMPs as Biomarkers

*MMP-2 and MMP-9*: In a study to assess the prognostic value of tissue MMP-2 in predicting prostate cancer outcomes, MMP-2 was found to be expressed by both cancer cells (70.0% cases) and stromal cells (75.9% cases); but in multivariate analyses, after adjusting for Gleason score, TNM stage, and initial serum prostate-specific antigen (PSA), only MMP-2 expression in >50% of malignant epithelial cells was associated with a shorter disease-free survival [50]. Using *in situ* hybridization, Wood *et al*. found that MMP-2 expression was an independent predictor of poor post-prostatectomy outcomes [29]. The prognostic role of MMP-9 is more controversial. Ozden *et al*. found that MMP-9 positivity in normal glands was correlated with lower Gleason scores and earlier stage at presentation [27]. In a univariate analyses of 278 patients with organ-confined prostate cancer, among MMP-2, -3, -7, -9, -13, and -19, higher expression levels of MMP-9 had a protective effect in terms of overall survival [51]. But in other studies, MMP-9 expression level by either stromal or cancer cells was not associated with prostate cancer disease-free survival [46] or biochemical recurrence [52]. It is not known whether the discrepancy was due to different detection methods used in the studies.

Serum MMP-2 and MMP-9 levels were shown to be significantly increased in prostate cancer patients compared to controls [53]. Among prostate cancer patients, those with metastatic disease had significantly higher plasma levels of MMP-2 and -9 than patients with localized disease [31,36]. In addition, serum MMP-2 levels correlated with the clinical course of prostate cancer with bone metastasis [31]. In addition to serving as a prognostic marker, plasma activity of MMP-2 and/or MMP-9, in association with PSA, may play a role in diagnosis and monitoring of therapy. Urinary MMP-9 activity was significantly higher in the urine samples from prostate cancer patients compared with controls [54]. Activity of both plasma MMP-2 and MMP-9 were significantly decreased in metastatic patients after therapy [36]. In addition, it has been shown that MMP-2 and MMP-9 levels in radical prostatectomy specimens were significant predictors of cancer recurrence [55]. Thus MMP-2 and MMP-9 expression in the tissue and body fluids may be of diagnostic, prognostic and predictive value in the detection and/or clinical monitoring of disease progression and therapeutic efficacy in patients with prostate cancer.

*MMP-7*: Epithelial cells in primary human prostate cancer express elevated levels of MMP-7 [43,47,56]. However the amount and proportion of the active and pro-enzyme forms of MMP-7 varies between cancers, and there is no correlation between extent of immunohistochemical MMP-7 expression with Gleason grade [47]. In a study investigating the serum levels of various MMPs in relation to the invasiveness of the prostate cancer, high circulating serum MMP-7 was associated with prostate cancer distant metastases [48], but the prognostic role of MMP-7 is not clear.

*MMP-15 and MMP-26*: In a comprehensive survey of the expression of various MMPs, serine protease families, and their natural inhibitors in human prostate cancer and benign prostate specimens, expression of several MMPs (MMP-10, -15, -24, -25 and -26) was increased in malignant tissue compared to benign prostate tissue [38]. Among them, the expression of MMP-15 and MMP-26 correlated positively with Gleason score, but the biology and prognostic potential of these two MMPs need to be further investigated in prostate cancer.

*TIMP-1*: Biochemical analysis, *in situ* hybridization and immunohistochemistry studies have clearly shown that TIMP-1 expression is lost in the tumor tissue of prostate adenocarcinoma cells and many other solid tumors [29,60]. It is thus contradictory that circulating TIMP-1 is elevated in multiple malignancies and that elevated circulating TIMP-1 levels have been associated with poor prognosis [147,148,149,150,151,152,153,154], given the predicted role of TIMP-1 as an inhibitor of metastasis. In prostate cancer, Jung et al. reported increased levels of TIMP-1 in the plasma of patients with metastatic disease compared to those of patients with organ-confined cancer or with benign prostatic hyperplasia [26,155]. Our group has reported that overall survival was significantly shorter in metastatic CRPC patients with higher plasma TIMP-1 expression [49]. So far, it is not yet known what contributes to elevated circulating TIMP-1. One should be cautious that it is the elevated levels of TIMP-1 in circulation that correlate with poorer prostate cancer prognosis, not necessarily corresponding tissue levels of TIMP-1 in tumor sites.

In summary, there appears to be a trend of increased expression of MMP-2, -7, -9 and TIMP-1 in the blood and increased expression of MMP-2, -7, -14, -15 and -26 tissue during malignant progression of prostate cancer, but more carefully designed perspective studies are needed to determine the clinical utility of these MMPs as biomarkers in prostate cancer. In addition, one must understand that elevated levels of such enzymes simply reflect their deregulated expression during prostate cancer progression. The biological roles of these MMPs along with deregulated expression of TIMPs remain to be further elucidated by functional studies. In some cases, elevated MMP expression may be a consequence of, rather than a driving mechanism for, prostate cancer progression. In the past, most biomarker studies have focused on the role of single MMPs on cancer progression. In the future it will be of interest to develop comprehensive MMP/TIMP expression profiles (or MMP/TIMP signatures) that correlate with disease progression or drug responses, which is particularly pertinent for patient stratification in the clinical development of MMPIs.

### 4.2. MMPs as Therapeutic Targets

Given the considerable evidence implicating increased MMP activity in various aspects of cancer progression, inhibiting MMPs initially seemed to be a promising therapeutic approach. In addition, MMPs appeared to be ideal drug targets—they are disease-associated, extracellular enzymes with a dependence on zinc for activity. For this reason, many MMP inhibitors (MMPIs) were designed and tested in animal models and in human clinical trials.

Although extensive studies have been conducted on the potential clinical use of MMPIs in cancer, only limited amounts of data have been reported in prostate cancer patients. The small synthetic MMPIs, batimastat and marimastat, were forerunners for clinical application of treating metastatic tumors. The compounds potently inhibit MMPs by reversibly chelating the zinc atom at active site of MMPs. Marimastat (BB2516) was the first MMPI to complete phase I and phase II trials in prostate cancer patients. In a phase I study, there was a 58% “response rate” (defined as no increase in serum PSA over the course of the study plus a partial response defined as 0%–25% decrease in serum PSA in 4 weeks) using a dose of greater than 50 mg twice daily, which was generally well tolerated [158]. In a phase II study, among thirty-nine patients that were treated, a significant decrease in PSA slope was shown in the 20 mg group when compared with the 5 mg group. However, increasing musculoskeletal toxicity was associated with higher doses. The study was discontinued for the two highest dose levels due to toxicity. Another MMPI, batimastat (BB-094), inhibited invasion of DU145 cells in Matrigel and in a murine diaphragm invasion assay [159]. Studies reported an inhibitory effect of batimastat on Mat-LyLu cancer cells *in vitro* and on tumor growth in the orthotopic cancer R3327 Dunning tumor rat model [160]. But there are no data available on the clinical evaluation of this compound.

Despite promising pre-clinical and early clinical trial data, first generation MMPIs failed to succeed in phase III trials, which dampened enthusiasm for this class of drugs [14]. The rationale behind the development of MMPIs was a basic assumption that MMPs contributed to key malignant activities in cancer. However, more recent studies have led to a rethinking of the potential roles of MMPs in cancer progression, since multiple mouse studies have shown that MMPs can also be beneficial. For instance, MMP-3, -8, -9 and -12 knockout mice have all revealed a role for these enzymes that may be considered as anti-cancer [161,162,163,164]. Rather than simply labeling specific MMP as “good” or “bad”, it is more helpful to evaluate their function in particular cellular contexts. Therefore, there is more need to determine the role of specific MMPs in specific stages of tumor progression, as well as to develop animal models that recapitulate the behavior of cancer in humans. Last but not least, in preclinical models, MMP inhibition was generally initiated at early stages of disease and maintained throughout tumor progression. Evaluation of clinical trial data also suggests that patients with early stage disease would benefit most from MMPIs, which argues that we need to reconsider the best timing of treatment and optimize patient selection criteria. Currently, the therapeutic role of MMPIs in cancer remains uncertain, but the lessons learned from first generation MMPI development will be invaluable in designing next generation MMPIs.

## 5. Conclusions

There has been an overwhelming amount of evidence implicating the critical roles of MMP family members in various aspects of prostate cancer progression. However, we still do not thoroughly understand the unique roles of each MMP and TIMP in the complex cancer microenvironment, as evidenced by the clinical failure of first generation MMPIs. As we discussed, many MMPs and TIMPs may originate from malignant cells themselves as well as from their surrounding stroma, and the interaction between tumor and stromal cells or components may mutually modify the pattern of the MMP expression. Therefore, researchers should design studies to more closely mimic *in vivo* scenarios in order to achieve the accurate knowledge of function and regulation of this important family of proteins. In addition, MMPs and TIMPs could affect cancer progression through multiple mechanisms and sometimes even exert both beneficial and harmful effects at the same time. We must also take extra caution when targeting MMPs, in order to only inhibit their detrimental effects. Overall, many serum and tissue MMPs are overexpressed and are of significant prognostic and predictive values in prostate cancer. Further studies should be carried out to incorporate these serum and tissue biomarkers into clinical trials to identify the optimal patient cohorts who will most likely to benefit from future MMPI and other therapies.
